# An Advertising Department

**Published:** 1876-03

**Authors:** 


*An Advertising Department—From a Colaborator.*—"We are glad to note the suggestion in your last, in regard to the importance of the advertising department in a medical journal. We trust that you will push up this department, and that your readers will aid you by drawing the attention of druggists and others to the houses which advertise with you. In these times of rapid development and discovery, the advertising department of a legitimate medical journal is of essential importance to the medical practitioner, as it constitutes the only safe medium through which they can obtain proper and reliable information. We trust that you will solicit advertisements from the best and most reliable houses, that your readers may know whom to recommend to our home druggists. The good work has commenced in our town. We are glad to say that a number of our druggists have supplied themselves from the house of J. M. Gordon, of Cincinnati, whose card you publish; also, one has supplied himself with Ayers' Hernial Truss from having seen it advertised in the RECORD, and the physicians have found it as recommended—a most valuable truss. We will further say, that the long and continued card of Codman & Shurtleff in the RECORD, has secured them many patrons in this city. Billups, Clapp & Co. are also gaining friends here. In this we are encouraged to continue our efforts for our favorite—THE RECORD—and shall urge our druggists and merchants to patronize the houses who advertise in it. We are favorably impressed with the advertisement of W. H. Schiefflin & Co. in your last, but his address is not given—an oversight we presume. We are pleased with your Book Notices—continue them. Physicians throughout this section are beginning to come out of the poverty caused by the war, and their libraries being scanty, they would like to see reviews of all new and important works. On this point you will hear from us, perhaps, again, as we propose to give special attention to the advertising interests of THE RECORD in our locality."

[NOTE.—The above, from one of our most active colaborers, we publish as evincing the growing interest which is taken in our enterprise. Let others emulate his example, and let druggists and manufacturing houses everywhere observe the importance of THE RECORD as an advertising medium. We thank our friend for his efforts in our behalf, and promise to do our best to carry out his suggestions. The address of W. H. Schiefflin & Co. is 170 William street, New York.—ED.]

